# A chromosome-length genome assembly and annotation of blackberry (*Rubus argutus*, cv. “Hillquist”)

**DOI:** 10.1093/g3journal/jkac289

**Published:** 2022-11-04

**Authors:** Tomáš Brůna, Rishi Aryal, Olga Dudchenko, Daniel James Sargent, Daniel Mead, Matteo Buti, Andrea Cavallini, Timo Hytönen, Javier Andrés, Melanie Pham, David Weisz, Flavia Mascagni, Gabriele Usai, Lucia Natali, Nahla Bassil, Gina E Fernandez, Alexandre Lomsadze, Mitchell Armour, Bode Olukolu, Thomas Poorten, Caitlin Britton, Jahn Davik, Hamid Ashrafi, Erez Lieberman Aiden, Mark Borodovsky, Margaret Worthington

**Affiliations:** School of Biological Sciences, Center for Bioinformatics and Computational Genomics, Georgia Tech, Atlanta, GA 30332, USA; Department of Horticultural Science, North Carolina State University, Raleigh, NC 27607, USA; The Center for Genome Architecture, Baylor College of Medicine, Houston, TX 77030, USA; Department of Computer Science, Center for Theoretical Biological Physics, Rice University, Houston, TX 77030, USA; Department of Genetics, Genomics and Breeding, NIAB-EMR, East Malling, Kent, UK; Natural Resources Institute, University of Greenwich, Medway Campus, Chatham Maritime, Kent, UK; Wellcome Sanger Institute, Hinxton, Cambridge CB10 1SA, UK; Owlstone Medical Ltd, Cambridge CB4 0GJ, UK; Department of Agriculture, Food, Environment and Forestry (DAGRI), University of Florence, Florence, Italy; Department of Agriculture, Food and Environment, University of Pisa, Pisa, Italy; Department of Agricultural Sciences, Viikki Plant Science Centre, University of Helsinki, 00790 Helsinki, Finland; Department of Agricultural Sciences, Viikki Plant Science Centre, University of Helsinki, 00790 Helsinki, Finland; Department of Molecular and Human Genetics, Baylor College of Medicine, The Center for Genome Architecture, Houston, TX 77030, USA; Department of Molecular and Human Genetics, Baylor College of Medicine, The Center for Genome Architecture, Houston, TX 77030, USA; Department of Agriculture, Food and Environment, University of Pisa, Pisa, Italy; Department of Agriculture, Food and Environment, University of Pisa, Pisa, Italy; Department of Agriculture, Food and Environment, University of Pisa, Pisa, Italy; USDA-ARS, National Clonal Germplasm Repository, Corvallis, OR 97333, USA; Department of Horticultural Science, North Carolina State University, Raleigh, NC 27607, USA; Department of Biomedical Engineering, Center for Bioinformatics and Computational Genomics, Georgia Tech, Atlanta, GA 30332, USA; Department of Horticulture, University of Arkansas, Fayetteville, AR 72701, USA; Department of Entomology and Plant Pathology, University of Tennessee, Knoxville, TN 37996, USA; Pairwise, Durham, NC 27701, USA; Pairwise, Durham, NC 27701, USA; Department of Molecular Plant Biology, Norwegian Institute of Bioeconomy Research, N-1431 Ås, Norway; Department of Horticultural Science, North Carolina State University, Raleigh, NC 27695, USA; Department of Computer Science, Center for Theoretical Biological Physics, Rice University, Houston, TX 77030, USA; Department of Molecular and Human Genetics, Baylor College of Medicine, The Center for Genome Architecture, Houston, TX 77030, USA; UWA School of Agriculture and Environment, The University of Western Australia, Crawley, WA 6009, Australia; Broad Institute of MIT and Harvard, Cambridge, MA 02139, USA; Shanghai Institute for Advanced Immunochemical Studies, ShanghaiTech, Pudong 201210, China; Department of Biomedical Engineering, School of Computational Science and Engineering, Center for Bioinformatics and Computational Genomics, Georgia Tech, Atlanta, GA 30332USA; Department of Horticulture, University of Arkansas, Fayetteville, AR 72701, USA

**Keywords:** *Rubus*, blackberry, Rosaceae, Rosoideae, biennial flowering, annual flowering, primocane-fruiting, chromosome-length genome assembly, Hi-C, annotation, repetitive content

## Abstract

Blackberries (*Rubus* spp.) are the fourth most economically important berry crop worldwide. Genome assemblies and annotations have been developed for *Rubus* species in subgenus *Idaeobatus*, including black raspberry (*R. occidentalis*), red raspberry (*R. idaeus*), and *R. chingii*, but very few genomic resources exist for blackberries and their relatives in subgenus *Rubus*. Here we present a chromosome-length assembly and annotation of the diploid blackberry germplasm accession “Hillquist” (*R. argutus*). “Hillquist” is the only known source of primocane-fruiting (annual-fruiting) in tetraploid fresh-market blackberry breeding programs and is represented in the pedigree of many important cultivars worldwide. The “Hillquist” assembly, generated using Pacific Biosciences long reads scaffolded with high-throughput chromosome conformation capture sequencing, consisted of 298 Mb, of which 270 Mb (90%) was placed on 7 chromosome-length scaffolds with an average length of 38.6 Mb. Approximately 52.8% of the genome was composed of repetitive elements. The genome sequence was highly collinear with a novel maternal haplotype-resolved linkage map of the tetraploid blackberry selection A-2551TN and genome assemblies of *R. chingii* and red raspberry. A total of 38,503 protein-coding genes were predicted, of which 72% were functionally annotated. Eighteen flowering gene homologs within a previously mapped locus aligning to an 11.2 Mb region on chromosome Ra02 were identified as potential candidate genes for primocane-fruiting. The utility of the “Hillquist” genome has been demonstrated here by the development of the first genotyping-by-sequencing-based linkage map of tetraploid blackberry and the identification of possible candidate genes for primocane-fruiting. This chromosome-length assembly will facilitate future studies in *Rubus* biology, genetics, and genomics and strengthen applied breeding programs.

## Introduction

Blackberries (*Rubus* spp.) are specialty fruits in the Rosoideae subfamily of Rosaceae, which are prized for their sweet, juicy berries that have a delicate aroma and a deep black color. The global blackberry industry has experienced rapid growth and change during the past 2 decades (Strik *et al.* 2007). Americans spent just over $656 million on blackberries during 2020, a 17% increase over the previous year (Produce Market Guide 2022). This growth has been driven by increased consumer demand, advanced production methods, year-round product availability, and new cultivars.

The *Rubus* genus likely has a North American origin and is divided into 12 subgenera (Focke 1910; Carter *et al.* 2019). Other economically important crops in the genus *Rubus* include red raspberries (*Rubus idaeus*) and black raspberries (*Rubus occidentalis*), both of which are diploid species belonging to subgenus *Idaeobatus.* In contrast, blackberries belong to subgenus *Rubus* and range from diploid to 12x (2n = 2x = 14 to 2n = 12x = 84). Species belonging to subgenus *Rubus* are believed to have diverged from other subgenera, including *Idaeobatus*, *Chamaebatus*, *Cylactis*, *Dalibardastrum*, and *Malachobatus*, approximately 15–20 MYA (Carter *et al.* 2019). Cultivated blackberries are not assigned a specific epithet because most cultivars have several species in their ancestry (Clark *et al.* 2007). In North America, erect and semierect blackberries grown for fresh-market production are bred at the tetraploid (2n = 4x = 28) level and are composed mostly of species native to the Central and Eastern United States, including *R. allegheniensis*, *R. argutus*, and *R. trivialis.* Processing cultivars with trailing growth habit are typically bred at higher ploidy levels (primarily 2n = 6x/7x = 42/49), and are most closely related to the Western North American blackberry species *R. ursinus* (Finn and Clark 2012).

**Fig. 1.**
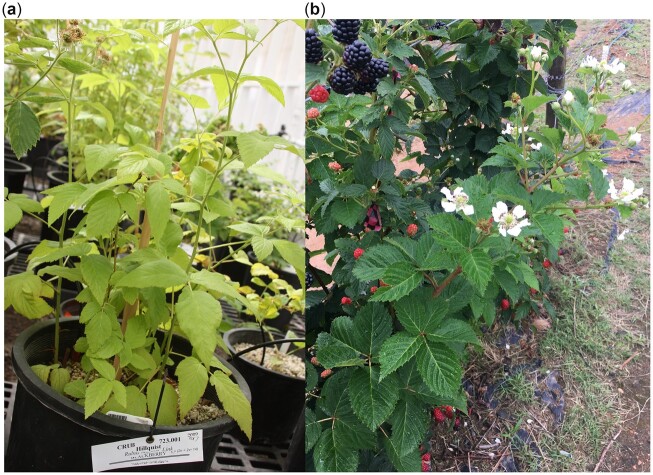
a) Hillquist blackberry (PI 553951) at the USDA National Clonal Germplasm Repository and (b) a primocane-fruiting blackberry with ripe fruit on second-year canes (floricanes) and flowers on the tip of a first-year cane (primocane).

*Rubus* plants are unusual among fruit crops because they typically have perennial crowns and root systems and biennial canes. First-year canes, which are usually vegetative, are called primocanes, while second-year canes that have overwintered are called floricanes. Floral initiation typically begins on primocanes in short days during the autumn, with flowers and fruits developing on floricanes the following spring (Williams 1959; Takeda *et al.* 2003; Sønsteby and Heide 2008). Raspberry and blackberry cultivars with this customary flowering trait are described as floricane- or biennial-fruiting. Primocane- or annual-fruiting red raspberry cultivars that initiate flowers in the early summer and produce fruit on the tip portion of primocanes or primocane branches during the late summer and autumn (Fig. 1) were first developed in the 1950s and 1960s (Keep 1988), with primocane-fruiting blackberries first commercially released in the early 2000s (Clark *et al.* 2005). Primocane-fruiting cultivars differ from traditional floricane-fruiting types in that they have no short-day requirement for flower induction and low-temperature requirement for flower emergence. Primocane-fruiting raspberries and blackberries have grown in economic importance over the past 2 decades because they confer several advantages for growers. The primocane crop is typically distinctly later than the floricane crop, which allows for season extension and the possibility for “double-cropping” by producing a floricane crop followed by a primocane crop from the same plant in each year. Furthermore, primocane-fruiting allows for production in an expanded geographical area, including tropical areas where there would be insufficient chilling hours for floricane cultivars, and regions where winter injury to canes is problematic (Clark 2008).

The only known source of primocane-fruiting in tetraploid blackberry cultivars is a recessive allele from the wild diploid accession “Hillquist” (*R. argutus*; PI 553951; Fig. 1). “Hillquist” was initially discovered in Ashland, VA by L.G. Hillquist, who noticed that some of the wild blackberries growing in his backyard had an unusual fruiting habit. The accession was later donated to the New York State Agricultural Experiment Station by Mrs Hillquist in 1949 (GRIN 2022). “Hillquist” was first used as a male parent in crosses with the tetraploid, floricane-fruiting blackberry cultivar “Brazos” in 1967, but the first primocane-fruiting cultivars, “Prime-Jim” and “Prime-Jan” were not released until nearly 40 years later (Clark *et al.* 2005; Clark 2008). Since then, many public and private blackberry breeding programs have accessed this germplasm, and “Hillquist” is in the pedigree of many important floricane-fruiting and primocane-fruiting cultivars grown around the world.

Despite their economic importance, very few genomic resources are available for blackberries compared with other fruit crops. Pseudo-chromosome level genome assemblies are available for over 20 Rosaceae crops, including apple (*Malus × domestica*) (Velasco *et al.* 2010; Daccord *et al.* 2017; Zhang *et al.* 2019), peach (*Prunus persica*) (Verde *et al.* 2013), and Asian pear (*Pyrus pyrifolia*) (Gao *et al.* 2021). Within the Rosoideae subfamily, which is characterized by a base chromosome number of x = 7, there are high-quality genome assemblies available for rose (*Rosa chinensis*) (Raymond *et al.* 2018) and diploid (*Fragaria vesca*) (Shulaev *et al.* 2011; Edger *et al.* 2018) and octoploid (*F. × ananassa*) (Edger *et al.* 2019) strawberry. The first *Rubus* genome sequenced was a highly homozygous diploid black raspberry selection, ORUS 4115-3 (VanBuren *et al.* 2016, 2018; Jibran *et al.* 2018). More recently, chromosome-length assemblies have been published for the red raspberry cultivar “Anitra” (Davik *et al.* 2022) and *R. chingii* (Wang *et al.* 2021). To date, however, the only published *Rubus* genome assemblies are for species in subgenus *Ideaobatus*, and there is no genome sequence data for any close relatives of cultivated blackberries in subgenus *Rubus* in public databases.

Here, we present a chromosome-length genome assembly and annotation of the diploid *R. argutus* accession “Hillquist.” “Hillquist” was chosen for the assembly because it is the original source of the primocane-fruiting used in cultivated tetraploid blackberries and is now represented in the pedigree of public and private blackberry breeding germplasm around the world. The *R. argutus* assembly was produced using Pacific Biosciences (PacBio) long-read single-molecule real-time (SMRT) sequencing and scaffolded using high-throughput chromosome conformation capture (Hi-C) sequence data. The full assembly is 298.2 Mb in length, with 270.0 Mb (90.1%) assigned to seven scaffolds with an average length of 38.6 Mb. Repetitive elements were predicted to make up 52.8% of the genome, with *Gypsy* superfamily lineages accounting for the largest fractions of long-terminal repeat (LTR)-retrotransposable elements (REs). The computational annotation was performed with support of RNA-sequencing (RNA-seq) and Iso-seq data generated from root tips and actively growing leaves and stems of primocane and floricanes. A total of 38,503 protein-coding genes were predicted from the genome, 72.2% of which were functionally annotated. The practical value of the *R. argutus* genome assembly and annotation was demonstrated by comparing the genome sequences of related Rosoideae species, anchoring the scaffolds to a novel modified genotyping-by-sequencing (GBS)-based (GBSpoly) linkage map of tetraploid blackberry, and identifying possible candidate genes for primocane-fruiting within a previously mapped locus.

## Materials and methods

### Plant material and genome size estimation

The *R. argutus* germplasm accession “Hillquist” (PI 553951), sourced from the USDA National Clonal Germplasm Repository (NCGR) was used for genome sequencing and assembly. Leaf material was harvested from a single plant of this cultivar grown in the greenhouse at the USDA-NCGR, in Corvallis, Oregon for flow cytometry, DNA extraction, and PacBio, 10× Chromium, and Hi-C sequencing. “Hillquist” plants propagated by NCGR staff were sent to North Carolina State University (NCSU) and grown in a greenhouse. Tissue from root tips and actively growing leaves and stems from primocanes and floricanes for RNA sequencing and IsoSeq was obtained from plants grown at NCSU. Nuclear flow cytometry with DAPI staining was used to measure DNA content and estimate the genome size of *R. argutus* “Hillquist.” Flow cytometry was performed using young, unexpanded “Hillquist” leaves in biological triplicate with *Vinca major* as an internal standard.

### DNA extraction, library preparation, and sequencing

#### Pacific Biosciences

High molecular weight DNA was extracted from young, unexpanded leaves of *R. argutus “*Hillquist” using a modified cetyl trimethylammonium bromide method (Porebski *et al.* 1997). DNA quality was evaluated with Pulsed Field Gel Electrophoresis (BioRad, Hercules, CA, USA), and quantification was performed with a Qubit fluorometer (ThermoFisher Sci., Waltham, MA, USA). Genomic DNA was sheared to achieve fragments in the 15–40 kb size range using a 26-gauge blunt end needle (ThermoFisher UK Ltd HCA-413-030Y guanine–cytosine Syringe Replacement Parts 26 g, 51 mm) and 1 ml luer-loc syringe. The sheared DNA was then cleaned using 1× AMPure PB Beads before library preparation. Fragments were enzymatically repaired and used to construct a long read (20 kb) PacBio Sequel genomic library with a SMRTbell Template Prep Kit 1.0-SPv3 according to the manufacturer’s recommendations (Pacific Biosciences Inc., Menlo Park, CA, USA). The resulting SMRTbell templates were size selected using BluePippin electrophoresis (Sage Science Inc., Beverly, MA, USA) and template DNA ranging in size between 15 and 50 kb was sequenced in eight PacBio Sequel SMRT cells on a PacBio Sequel instrument at the NCSU Genomic Sciences Laboratory.

#### Hi-C and 10× Genomics

Five grams of young leaf tissue for Hi-C and 10× Genomics library preparation was collected from a “Hillquist” plant subjected to 48 h of darkness. An in situ Hi-C library was prepared following (Rao *et al.* 2014) and sequenced as 150 base pairs (bp) paired-end reads using the Illumina HiSeq4000 platform. 10× Genomics linked read libraries were made at the Wellcome Sanger Institute High-Throughput DNA Sequencing Centre by the Sanger Institute R&D and pipeline teams using the Chromium Genome Reagent Kit (v2 Chemistry) following the manufacturer’s recommended protocol. These libraries were then sequenced on Illumina NovaSeq 6000 platforms at the Wellcome Sanger Institute High-Throughput DNA Sequencing Centre.

### Genome sequence assembly

A contig-scale assembly was generated with PacBio sequence data using the FALCON and FALCON-Unzip software applications (Chin *et al.* 2016). Error correction on the phased assembly was performed using the Arrow consensus model in the PacBio GenomicConsensus package following default parameters. The *k-mer* distribution of unassembled, corrected PacBio reads for “Hillquist” showed a bimodal distribution, indicating high heterozygosity. Therefore, the Purge Haplotigs pipeline was used to curate the heterozygous diploid genome assembly and resolve under-collapsed heterozygosity by identifying syntenic pairs of contigs and moving one to a haplotig pool (Roach *et al.* 2018). Hi-C data were aligned to the Purge Haplotigs draft assembly using Juicer v1.6.2 (Durand *et al.* 2016). A candidate assembly and contact maps visualizing the alignments with respect to the draft and the new reference were built using the 3D de novo assembly (3D-DNA) pipeline (Dudchenko *et al.* 2017), and the genome was reviewed and polished using Juicebox Assembly Tools (https://github.com/aidenlab/Juicebox). Chromosome nomenclature and orientation were assigned following Fragaria conventions (Shulaev *et al.* 2011).

Heterozygosity and genome size were estimated by analysis of the *k-mer* count histogram generated with 10× Chromium Illumina reads using the online version of GenomeScope (GenomeScope, RRID: SCR_017014; Vurture *et al.* 2017). The *k-mer* profile measures how often substrings of length *k* occur in raw short read sequencing reads. GenomeScope fits a mixture model of 4 evenly spaced negative binomial distributions to the *k-mer* profile to measure the relative abundances of heterozygous (unique) and homozygous (2-copy) sequences to estimate heterozygosity and estimates genome size by normalizing the observed *k-mer* frequencies to the average coverage value for homozygous sequences, excluding likely sequencing errors.

### Synteny with Rosoideae genomes

Synteny of the “Hillquist” genome to the other publicly available Rosoideae genome sequences [*R. idaeus* “Anitra” (Davik *et al.* 2022), *R. chingii* (Wang *et al.* 2021), *R. occidentalis* (VanBuren *et al.* 2018), *F. vesca* “Hawaii 4” (Edger *et al.* 2018), and *Rosa chinensis* “Old Blush” (Raymond *et al.* 2018)] was determined with MUMmer4 (Marçais *et al.* 2018) using default parameters. Data for the genomes were downloaded from the data repository on the Genome Database for Rosaceae (https://www.rosaceae.org; Jung *et al.* 2019), and the associations revealed were plotted using R following Davik *et al.* (2022).

### Linkage map of autotetraploid blackberry

A mapping population consisting of 119 F_1_ progeny from the cross A-2551TN × APF-259TN (Supplementary Fig. 1) were used to generate a maternal haplotype map. Full methods and results for map construction are provided in Supplementary File 1. Multiplexed NGS-based reduced representation sequencing libraries for parents and progeny were prepared following the GBSpoly protocol optimized for heterozygous and polyploid genomes (Wadl *et al.* 2018; Mollinari *et al.* 2020) and sequenced on the HiSeq 2500 (Illumina, San Diego, CA, USA) and the SP flow cell of the NovaSeq 6000 (Illumina, San Diego, CA, USA) system at the Genomic Sciences Laboratory at NCSU to generate 615.4 million sequencing reads after demulitplexing and quality filtering. Raw Fastq files were processed and filtered with the ngsComposer (Kuster *et al.* 2021) pipeline (https://github.com/bodeolukolu/ngsComposer) and were aligned to the black raspberry (VanBuren *et al.* 2018) and “Hillquist” genomes using Burrows–Wheeler Aligner (BWA)–MEM (https://github.com/lh3/bwa). The GBSapp pipeline (https://github.com/bodeolukolu/GBSapp), which integrates original and third-party tools (bwa, samtools, picard, bcftools, GATK, java, R-ggplot2, and R-AGHmatrix), was used for variant calling and filtering. Only single dose markers segregating in A-2551TN were used to construct the haplotype-resolved maternal linkage map. Markers that had less than 5% missing data, were heterozygous in A-2551TN (0/0/0/1 *×* 0/0/0/0), and segregated in a 1:1 ratio in the progeny were used to create a maternal linkage map in JoinMap 4.1 (Van Ooijen 2006).

### *Analysis of repetitive conten*t

The repetitive component of the “Hillquist” genome was analyzed using both structural- and clustering-based characterization analyses. Structural-based results were compared with those of the other 4 Rosaceae species (*F. vesca*, *Potentilla micrantha*, *P. persica*, and *M. domestica*). The data of the other 4 Rosaceae species were retrieved from the National Center for Biotechnology Information (NCBI) database (NCBI, Washington, USA, https://www.ncbi.nlm.nih.gov/) and the GigaScience GigaDB repository (Supplementary Table 1). The quality of the “Hillquist” paired-end Illumina reads was inspected using FastQC v0.11.5 (Andrews 2010), and Illumina adapters and low-quality regions were removed using Trimmomatic v0.39 (Bolger *et al.* 2014) with the following parameters: ILLUMINACLIP: 2:30:10; LEADING: 3; TRAILING: 3; SLIDINGWINDOW: 4:15; CROP: 90; MINLEN: 90. Duplicated reads were removed using the prinseq-lite.pl script v0.20.4 with -derep 1 (Schmieder and Edwards 2011). Organellar sequences were removed from the datasets by mapping against an ad hoc prepared set of chloroplast genomes of *F. vesca* (NCBI JF345175.1), *M. domestica* (NCBI MK434916.1), *P. micrantha* (NCBI HG931056.1), *P. persica* (NCBI HQ336405.1), and *Rubus leucanthus* (NCBI MK105853.1) and mitochondrial genomes of *M. domestica* (NCBI NC_018554.1) and *Prunus avium* (NCBI MK816392.2) using CLC-BIO Genomic Workbench v9.0.4 (CLC-BIO, Aarhus, Denmark) with the following parameters: mismatch cost 1; insertion cost 1; deletion cost 1; length fraction 0.9; similarity fraction 0.9. All matching sequences were considered putatively belonging to organellar genomes and subsequently removed.

#### Clustering-based characterization of repeats

A clustering characterization of the repetitive component of the “Hillquist” genome was performed using RepeatExplorer2 (Novák *et al.* 2020) with default parameters with a random set of 1,000,000 paired-end sequences. To reduce the number of unknown retrotransposon clusters, BLASTN and tBLASTX (Altschul *et al.* 1990) analyses were performed using Basic Local Alignment Search Tool (BLAST) v2.6.0 with default parameters against the libraries of the characterized Rosaceae full-length LTR-REs.

#### Full-length LTR-retrotransposon discovery and characterization analysis

The genome assemblies of “Hillquist,” *F. vesca*, *P. micrantha*, *P. persica*, and *M. domestica* were scanned for a structural identification of Class I full-length LTR-REs using LTRharvest v1.5.10 (Ellinghaus *et al.* 2008) with the following parameters: -minlenltr 100; -maxlenltr 6000; -mindistltr 1500; -maxdistltr 25000; -mintsd 5; -maxtsd 5; -similar 85; -vic 10; -motif tgca. The libraries of full-length LTR-REs were submitted to domain-based annotation by using DANTE v1.0.0, available on the RepeatExplorer Galaxy-based website (https://galaxy-elixir.cerit-sc.cz/). The annotation process was performed with default parameters using the REXdb of transposable element protein domains (Neumann *et al.* 2019) and a BLOSUM80 scoring matrix. The protein matches were filtered by significance using the parameters provided by the platform, and nested elements were manually removed. To reduce the number of uncharacterized full-length LTR-REs, we performed BLASTN and tBLASTX between uncharacterized elements and characterized elements in conjunction with the annotated contigs produced by the comparative clustering analysis.

### Gene prediction and annotation

#### RNA extraction, library preparation, and sequencing

Total RNA was extracted from 5 tissue types (root tips, as well as actively growing leaves and stems from both primocane and floricane canes of the same plant) for sequencing with RNA-Seq and Iso-Seq technologies using the Spectrum Plant Total RNA Kit (Millipore Sigma, Burlington, MA, USA) following the manufacturer’s protocol. Cane types were distinguished by the presence of trifoliate leaves on floricanes and pentifoliate leaves on primocanes. The purity and concentration of the extracted RNA was determined using a 2100 Bioanalyzer (Agilent Technologies, Santa Clara, CA, USA), and the integrity of the samples was determined using a Qubit 4.0 fluorimeter (Thermo Fisher Scientific, Waltham, MA, USA). Samples with an RNA integrity number value above 7.0 were submitted for subsequent sequencing. Two duplicate RNA-Seq libraries were produced for each tissue type and sequenced with an Illumina HiSeq X instrument at Scientific Operations core at the Wellcome Sanger Institute. Total RNA from the same 5 tissue samples were pooled and used for Iso-Seq library preparation. Standard PacBio Iso-Seq SMRTbell libraries were prepared by Genewiz (South Plainfield, NJ, USA) and one SMRT cell was sequenced with Sequel II. Full-length transcripts were identified using the Iso-Seq 3 application in SMRTLink 5.0. First, multiple reads of the same SMRTbell sequence or the subreads from the same polymerase read were combined to produce one high-quality circular consensus sequence (CCS). Next, the CCS reads were classified as full-length based on the presence of both complementary DNA primers and polyA tails in the reads. Full-length reads were further classified as chimeric or nonchimeric reads based on whether or not primers were found in the middle of the sequences. Finally, unpolished consensus isoforms were extracted using the iterative clustering and error correction algorithm and polished to obtain high-quality and low-quality isoforms.

#### Structural gene annotation

A repeat library of transposable element families was generated using RepeatModeler2 (Flynn *et al.* 2020). Repeat sequences, interspersed repeats, and low complexity DNA sequences were identified and soft-masked using RepeatMasker (Smit *et al.* 2013). Repeat masking was further refined using Iso-Seq transcript sequences. The representative Iso-Seq open reading frames (ORFs) supported by protein or RNA-Seq evidence were used to reduce the amount of repeat-masked coding sequence by unmasking the masked regions overlapping the Iso-Seq defined ORFs. RNA-Seq mapping originated intron hints were obtained by aligning paired RNA-Seq reads to the “Hillquist” genome using STAR (Dobin *et al.* 2013) with filters for the intron coverage value ≥3. Additionally, consensus high-quality Iso-Seq isoforms were aligned to the genome by GMAP (Wu and Watanabe 2005) with filters for ≥95% identity and ≥90% coverage. The longest ORF (lORF) was identified in each aligned transcript. In loci with overlapping isoforms, a single representative transcript with the longest lORF was selected thus making a set of nonoverlapping Iso-Seq isoforms. Transcripts with lORFs shorter than 300 nucleotides or with introns longer than 10,000 nucleotides were filtered out from this set. Protein hints to splice sites and translation initiation and termination sites were generated by ProtHint (Brůna *et al.* 2020) using proteins from the Plantae section of the OrthoDB v10 protein database (Kriventseva *et al.* 2019).

Genes were annotated using a protocol similar to BRAKER2 (Brůna *et al.* 2021), with additional integration of RNA-Seq and Iso-Seq data (Supplementary Fig. 2). GeneMark-ET (Lomsadze *et al.* 2014) with RNA-Seq intron hints was used to create a set of predicted genes. In this analysis, introns mapped with coverage ≥100 were used for initial parameter estimation. Genes predicted by GeneMark-ET were subsequently used as seed regions in ProtHint to generate protein hints. Next, protein and RNA-Seq hints were used together to predict genes with GeneMark-EP+ (Brůna *et al.* 2020). By default, GeneMark-EP+ directly uses protein hints generated by ProtHint. This hint set was extended by adding RNA-Seq intron hints. Introns found in the intersection of RNA-Seq and protein hints were added to GeneMark-EP+’s high-confidence hint set. Genes predicted by GeneMark-EP+ and ORFs from the set of nonoverlapping Iso-Seq isoforms were combined to create the new seed regions. In case of an overlap between the Iso-Seq and GeneMark-EP+ defined seeds, the Iso-Seq seed was selected if its ORF was >50 nt longer than the GeneMark-EP+ seed. GeneMark-EP+ was then run on the genome with updated repeat-masking and protein hints delivered by the second iteration of ProtHint. Again, RNA-Seq hints were added to the hints set in the same way as described for the first GeneMark-EP+ run. GeneMark-EP+ predictions fully supported by mapped Iso-Seq transcripts or protein hints were selected for the training of AUGUSTUS (Stanke *et al.* 2006). AUGUSTUS was run on the “Hillquist” genome sequence with refined repeat-masking and ProtHint proteins hints in agreement with the BRAKER2 protocol (Brůna *et al.* 2021) to generate the final gene predictions.

The predicted genes were categorized according to their support by external evidence. Multiexon transcripts were *fully supported* by Iso-Seq if all introns had support by at least a single Iso-Seq transcript. The supporting Iso-Seq transcript could not contain any additional introns, except in its 5’ and 3’ UTRs. Multiexon transcripts were fully supported by proteins or RNA-Seq if all their introns were supported by protein or RNA-Seq hints. Single exon transcripts were fully supported by Iso-Seq if a matching lORF was found in one of the Iso-Seq transcripts. Single-exon transcripts were fully supported by proteins if the start and stop codons were supported by protein hints. Transcripts *supported* by any evidence were required to have a part of their gene structure supported by an Iso-Seq, RNA-Seq, or a protein hint. The Benchmarking Universal Single-Copy Orthologs (BUSCO) (Seppey *et al.* 2019) toolkit was used to assess how many predicted *R. argutus* genes were coding for Universal Single-Copy Orthologs. Furthermore, we used Liftoff (Shumate and Salzberg 2021) to map annotated genes from *F. vesca* (annotation v4.0.a2; assembly v4.0.a1; Li *et al.* 2018) onto the *R. argutus* assembly.

#### Functional gene annotation

Putative gene function was determined through interrogation of the Swiss-Prot, Araport11, NCBI nr, Refseq, and TrEmbl protein databases with BLAST+ blastp-fast algorithm (Camacho *et al.* 2009) using the predicted protein-coding sequences of the 38,503 genes identified in the structural annotation as queries with an expectation value cutoff of 1e−6. BLAST+ analyses were executed using the Galaxy platform (Afgan *et al.* 2018) with locally installed databases except for Araport11, which was downloaded from The Arabidopsis Information Resource (TAIR, https://www.arabidopsis.org/). InterProScan v5 (Zdobnov and Apweiler 2001) was used to assign InterPro domains, and Gene Ontology (GO) terms to the predicted proteins. KEGG ortholog and KEGG pathway mapping were performed with BlastKOALA v2.2 (Kanehisa *et al.* 2016) and eggNOG-mapper v2 (Huerta-Cepas *et al.* 2017), respectively.

### Potential candidate genes for primocane-fruiting in blackberry

To explore candidate genes for the primocane-fruiting trait in blackberry, blackberry homologs of the *Arabidopsis* flowering time genes listed in FLOR-ID database were mined from the “Hillquist” genome sequence (Bouché *et al.* 2016). A previously mapped 11.2 Mb region (Castro *et al.* 2013) corresponding to *R. argutus* chromosome Ra02 at 25,901,374–37,085,204 bp was specifically targeted for potential primocane-fruiting candidate genes.

## Results and discussion

### Chromosome-length genome assembly

**Fig. 2.**
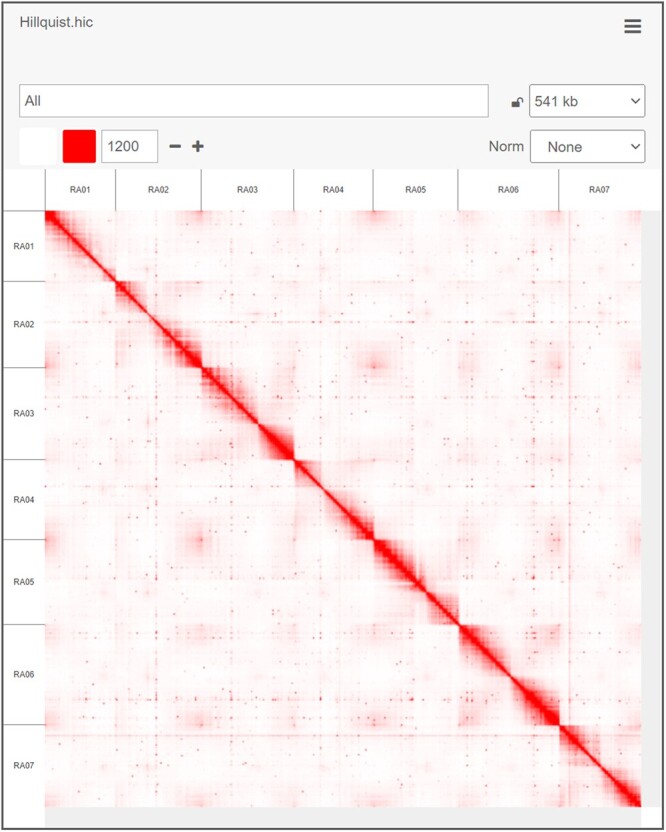
Hi-C interaction matrix for the “Hillquist” blackberry (*R. argutus*) assembly. An interactive version of this map is available at https://tinyurl.com/2eldso37.

A combined total of 3.8 million PacBio post-filtered reads with an average length of 6,824 bp were generated from the eight SMRT cells, resulting in a total of 25.9 Gb of sequence (∼77× Genome Coverage) (Supplementary Table 2). These reads were used to generate an initial FALCON-Unzip assembly comprised 374 Mb of sequence in 1,756 contigs with an N50 of 486 kb and a maximum contig length of 5.9 Mb. After Purge Haplotigs was used to resolve under-collapsed heterozygosity, the optimized assembly consisted of 297 Mb assigned to 811 primary contigs with a contig N50 of 650 Kb and a maximum contig length of 5.9 Mb. The Hi-C library was sequenced to produce 559,559,351 paired-end reads. Hi-C data were aligned to the Purge Haplotigs draft assembly to create a new 298 Mb assembly composed of 350 scaffolds with an N50 of 38.6 Mb and a maximum scaffold length of 45.5 Mb (Table 1 and Fig. 2). Among these Hi-C scaffolds, seven chromosome-length scaffolds with a total length of 270 Mb (90% of the 298 bp genome) corresponded directly to the seven *R. occidentalis* and *F. vesca* chromosomes (Supplementary Table 3).

**Table 1.** Summary statistics for the assembled *R. argutus* “Hillquist” genome.

Estimated genome size (flow cytometry)	337.4 Mb
Estimated genome size (25-mer)	298.06 Mb
Total assembly length	298.24 Mb
No. of scaffolds	350
No. of chromosomes	7
Size of sequence anchored on chromosomes	270 Mb
Maximum scaffold length	45.5 Mb
N50 scaffold length (bp)	38.6 Mb
Number of predicted protein-coding genes	38,503
Average coding sequence length	2,183 bp

### Genome size estimation

The nuclear flow cytometry generated estimate of the *R. argutus* genome size was 337.4 Mb (1C = 0.345 pg). This estimate falls within the reported range of other diploid species in subgenus *Rubus* (*R. hispidus*, *R. canadensis*, *R. trivialis*, *R. canescens*, and *R. sanctus*), which was between 1C = 0.295–0.375 pg (Thompson 1995; Meng and Finn 2002). Heterozygosity and genome size were also estimated by analysis of the *k-mer* count histogram generated with 10× Chromium Illumina reads using the online version of GenomeScope [GenomeScope, RRID: SCR_017014 (Vurture *et al.* 2017)]. The size and heterozygosity of the genome were estimated as 298.06 Mb and 1.04% (Supplementary Fig. 3). The *k-mer* based genome size estimate was within 172.4 kb of the Hi-C assembly length, which suggests that the genome was nearly complete. However, the flow cytometry estimate of *R. argutus* genome size was 337 Mb, indicating that 88.4% of the genome was incorporated in the assembly.

### Synteny with Rosoideae genomes

**Fig. 3.**
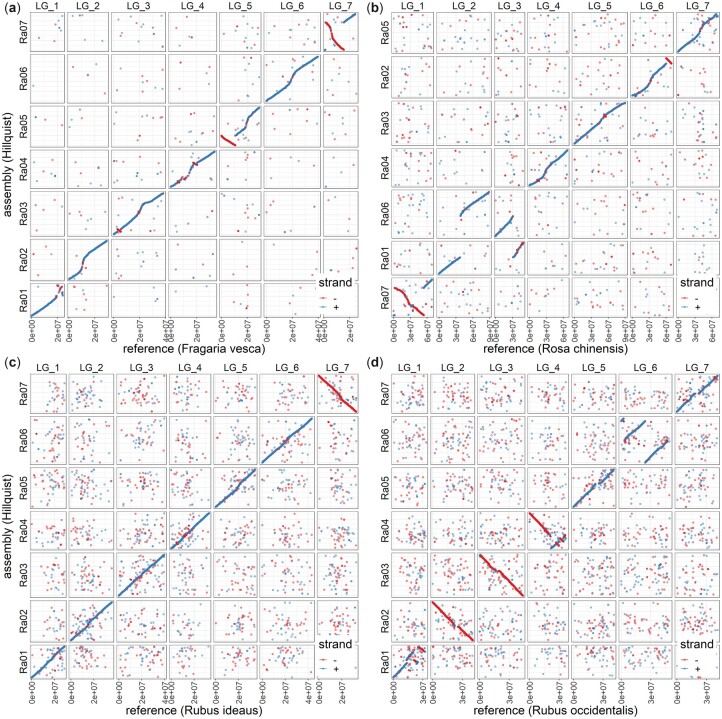
Whole-genome alignment plots between the “Hillquist” blackberry (*R. argutus*) genome assembly and the chromosome-length assemblies of (a) woodland strawberry (*Fragaria vesca* V. 4), (b) rose (*Rosa chinensis*), (c) red raspberry (*R. idaeus*), and (d) black raspberry (*R. occidentalis* v.3).

The “Hillquist” assembly showed a high degree of collinearity to the other Rosoideae genomes (Fig. 3; Supplementary Fig. 4). Collinearity to the genomes of *R. idaeus* “Anitra” and *R. chingii* was particularly high, with no large-scale rearrangements, translocations, or inversions observed across any of the 7 chromosomes when compared with these 2 species (Fig. 3c; Supplementary Fig. 4). As with the previously published comparison of the *R. idaeus* “Anitra” and *R. occidentalis* genome assemblies (Davik *et al.* 2022), several areas of noncollinearity were observed between the “Hillquist” and *R. occidentalis* genomes. The most notable areas of non-collinearity between the genomes were the large degree of rearrangement on one half of chromosomes 1 and 4 and the 2 large inversions originating from the same chromosomal breakpoint identified on chromosome 6 (Fig. 3d). Other authors (Davik *et al.* 2022) have suggested that these differences could be the result of errors in the assembly of the *R. occidentalis* genome, and the data presented here support that hypothesis. The pattern of rearrangements observed between the “Hillquist” genome and *F. vesca* “Hawaii 4” and *R. chinensis* “Old Blush” genomes were similar to those previously reported for the “Anitra” genome (Davik *et al.* 2022). Two large inversions on chromosomes 5 and chromosome 7 and with several smaller inversions on chromosomes 3 and 4 were observed between the “Hillquist” genome and that of *F. vesca* “Hawaii 4” (Fig. 3a). Two significant translocations were observed between chromosome 1 and chromosome 6 of the “Hillquist” and *R. chinensis* “Old Blush” genomes, along with small inversions on chromosomes 2 and 7 (Fig. 3b). These rearrangements reflect the evolutionary timescales since the *Rubus*, *Fragaria*, and *Rosa* ancestral genomes diverged from a common ancestor (Longhi *et al.* 2014).

### Linkage map of autotetraploid blackberry

The best available blackberry linkage map was constructed using 119 simple sequence repeat (SSR) markers developed from red raspberry and a blackberry expressed sequence tag library (Castro *et al.* 2013). Due to the paucity of markers, this SSR-based map contained large genetic regions with no marker coverage. The utility of the “Hillquist” genome sequence for use in fresh-market blackberry breeding was therefore assessed by anchoring the pseudo-chromosomes to a novel linkage map of the autotetraploid breeding selection, A-2551TN, from the University of Arkansas System Division of Agriculture Fruit Breeding Program. The linkage map consisted of 2,935 sequence-characterized markers that were identified using a modified GBS protocol (GBSpoly) that is robust for highly heterozygous and polyploid genomes. In total, 85.9% of quality filtered reads were mapped to unique positions and 2,022,664 polymorphic markers were identified when the “Hillquist” genome was used as a reference, while only 67.3% of reads mapped to unique positions and 1,811,617 polymorphic markers were discovered when the black raspberry genome was used as the reference (Supplementary Table 4).

**Fig. 4.**
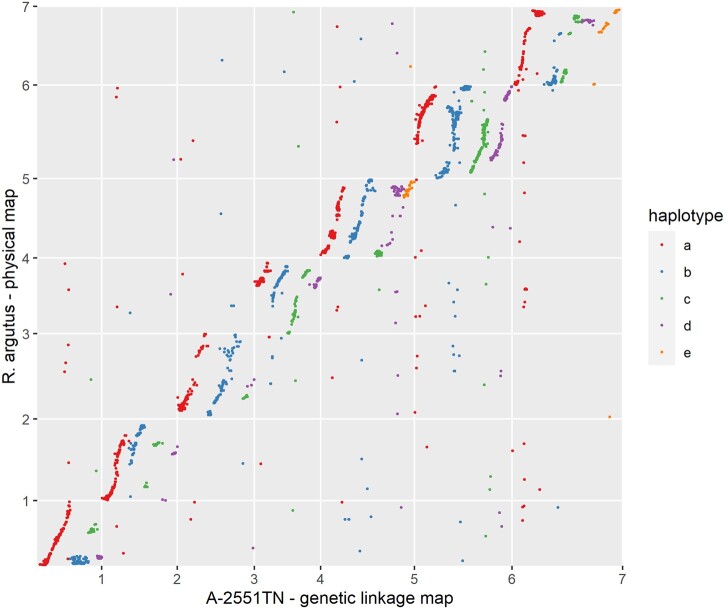
Comparison of the tetraploid A-2551TN maternal haplotype map with the “Hillquist” blackberry (*R. argutus*) physical map. As expected, 4 homologous linkage groups (haplotypes a–d) were identified for chromosomes Ra01, Ra02, Ra03, Ra04, and Ra06. Five homologous linkage groups (haplotypes a–e) corresponded to chromosomes Ra05 and Ra07. Based on the physical positions of the markers on chromosomes Ra05 and Ra07, it is likely that linkage groups 5c and 5e and 7b and 7d actually belong to the same haplotype of A-2551TN.

**Fig. 5.**
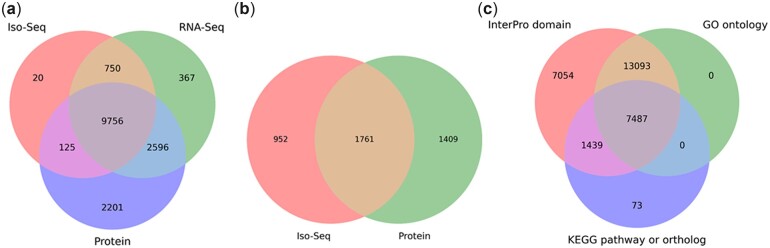
Predicted (a) multiexon transcripts and (b) single-exon transcripts fully supported by external evidence and (c) predicted transcripts with functional annotation matches.

Only single dose markers segregating in A-2551TN were used to construct the haplotype-resolved maternal linkage map, which was composed of 2,935 markers assigned to 30 linkage groups, with between 5 and 249 markers per linkage group. The total map length was 2,411.81 cM, with linkage groups ranging from 18.61 to 146.65 cM in length and an average of one marker every 0.82 cM (Supplementary Tables 5 and 6 and Fig. 5). The physical positions of the mapped markers on the “Hillquist” pseudo-chromosomes were used to identify 4 homologous linkage groups corresponding to 5 chromosomes (1, 2, 3, 4, and 6), and 5 homologous linkage groups corresponding to the remaining 2 chromosomes (5 and 7). The A-2551TN maternal haplotype map was strongly collinear with the “Hillquist” genome, with no major translocations or inversions (Fig. 4). While many of the linkage groups in the A-2551TN maternal haplotype map contained markers that aligned to physical positions across the length of each of the chromosomes, 10 linkage groups had markers aligned to physical positions spanning less than 10 megabase pairs (Mbp) in the “Hillquist” genome. Based on the physical positions of these markers on short linkage groups, it is likely that linkage groups 7b/7d and 5c/5e belong to the same haplotype of A-2551TN. Gaps in the linkage map can likely be attributed to the high inbreeding coefficients of A-2551TN (*F* = 0.100) and its progeny from the A-2551TN × APF-259TN cross (*F* = 0.099). The high percentage of reads mapped to unique positions on the “Hillquist” genome and the collinearity between the physical map of “Hillquist” and the A-2551TN maternal haplotype map validate the order and orientation of the Hi-C-based chromosome-length assembly of “Hillquist” and demonstrate its utility for genomic breeding research in polyploid fresh-market blackberries.

#### Clustering-based characterization of repeats

Of the 1 million “Hillquist” paired-end Illumina reads randomly selected for de novo clustering, 555,442 reads were processed by RepeatExplorer2 (Novák *et al.* 2020). Of these processed reads, 262,064 (47.2% of the genome) were considered singlets and did not fall into the category of repeated sequences according to the thresholds imposed by the program. The remaining 293,379 reads (52.8% of the genome) were characterized as repeats and grouped in 51,851 clusters, each of which represented a single repeat sublineage. One hundred and seventy-three clusters were classified as top clusters with a genome proportion greater than 0.01%, representing the most abundant repeat families. *Copia* and *Gypsy* superfamilies accounted for the largest fractions of the genome (10.51% and 23.44%, respectively; Table 2). In particular, *Athila*-related clusters were the most abundant. No DNA transposons, non-LTR elements, or satellite DNA were among the top clusters. The absence of DNA transposon and satellite DNA in top clusters indicates that these repeat types are scarce in the *Rubus* genome. Illumina reads related to these repeats were assembled in clusters accounting for less than 0.01% of the genome. Finally, 18.14% of the repetitive component remained unclassified.

**Table 2.** Classification of clusters produced by RepeatExplorer2 and proportion of repeat types in the genome of “Hillquist” (*R. argutus*).

Classification	Genome proportion (%)	Number of clusters
*Copia*	10.51	24
*Ale*	2.03	2
*Angela*	0.07	1
*Bianca*	5.78	12
*Ikeros*	0.86	2
*Ivana*	0.01	1
*SIRE*	0.96	3
*Tork*	0.8	3
*Gypsy*	23.44	45
*Chromovirus*	4.28	5
*Athila*	17.84	28
*Ogre/Tat*	1.32	12
rDNA	0.79	4
Unclassified	18.08	
Low/single	47.18	

#### Full-length LTR-retrotransposon discovery and characterization analysis

A total of 636 full-length LTR-REs were identified in the “Hillquist” genome assembly, with 217 and 409 LT-REs belonging to the *Gypsy* and *Copia* superfamilies, respectively (Supplementary Table 7). The number of full-length LTR-REs isolated from the other 4 genome assemblies of Rosaceae species varied from a minimum of 204 in *F. vesca* to a maximum of 2,662 in *M. domestica* (Supplementary Table 7). *Copia* elements were more abundant than *Gypsy* LTR-REs in “Hillquist” (1.9:1), *F. vesca* (2.7:1), and *P. persica* (4.5:1), while LTR-REs in *Copia* and *Gypsy* superfamilies were equally represented in *P. micrantha* (1:1), and *Gypsy* elements were slightly more abundant in *M. domestica* (0.7:1). The lineage level annotation of most elements revealed considerable quantitative and qualitative variability among the 5 species, with several lineages that were not detected in some species. However, it is possible that very ancient and rearranged elements may not have been identifiable through structural features due to the stringency of the parameters used in the identification process.

### Gene prediction and annotation

#### RNA extraction, library preparation, and sequencing

A total of 135,518,570 paired reads were generated from the 10 RNA-Seq libraries (2 duplicate libraries of 5 tissue types: root tips, and actively growing leaves and stems from both primocane and floricane canes of the same plant), with 9,457,856–20,119,374 paired reads per library. One SMRT cell with a library prepared from pooled RNA from the same 5 tissue samples was sequenced with Sequel II to generate a total of 5,959,439 polymerase reads with a mean length of 39,878 bp per read, an average insert length of 7,387 bp, and a mean subread length of 1,614. A total of 2,830,415 CCS reads with a mean length of 1,526 bp were generated from these reads. Finally, 185,699 and 290 polished high-quality and low-quality isoforms were generated from the IsoSeq data.

#### Structural gene annotation

One hundred and thirty-five megabase pairs (45.4%) of the “Hillquist” genome was repeat masked prior to structural annotation. The repeat length distribution is shown in Supplementary Fig. 6. Masking refinement based on aligned Iso-Seq transcripts unmasked 1.9 Mbp of the sequence at 7,257 distinct Iso-Seq loci. The final set of predicted genes contained 38,503 coding genes and, with counting alternative isoforms, 40,397 coding transcripts. A total of 13,364 of these transcripts were fully supported by Iso-Seq transcripts, while RNA-Seq data fully supported 13,469 transcripts, and 17,848 transcripts had full protein support (Fig. 5, a and b); 31,326 transcripts were partially supported by some evidence, and the remaining 9,407 transcripts were pure ab initio predictions. Transcripts in the unsupported group were rather short (average protein length 166 AA), with a large fraction (5,129; 55%) lacking any introns. Overall, 19,937 genes had full support from at least one of the external evidence types. The average length of proteins encoded by transcripts with at least one type of external evidence support was 400 AA; this set included 6,125 intronless transcripts. The 38,503 coding genes had an average length of 2,183 bp, containing an average of 3.4 introns per gene and median intron and exon lengths of 152 and 132 bp, respectively. Of these coding genes, 36,836 had no alternative isoforms, 1,466 had 2 isoforms, and 201 had 3 or more isoforms (Supplementary Fig. 7). The number of predicted genes in *R. argutus* was comparable to other *Rubus* genomes including *R. idaeus* (39,448; Davik *et al.* 2022), *R. chingii* (33,130; Wang *et al.* 2021), and *R. occidentalis* (34,545; VanBuren *et al.* 2018). Liftoff, using default parameters, mapped 21,480 genes (63% of genes in the *F. vesca* annotation v4.0.a2) and the mapped gene structures closely agreed with our predicted gene structures: 68% and 95% of mapped exons matched exons in our *R. argutus* annotation exactly and partially, respectively. In the predicted set of genes, 2,134 (91.7%) complete *R. argutus* genes orthologous to the BUSCO families were identified, along with 74 (3.2%) genes with partial match. A small fraction of the BUSCO families (5.1%) were not identified among the predicted *R. argutus* genes (Supplementary Fig. 8). These results suggest that the “Hillquist” assembly and the gene complement are 94.9% complete.

#### Functional gene annotation

Of the 40,397 transcripts predicted in the “Hillquist” genome, a total of 15,333 (37.96%), 22,713 (56.22%), 15,639 (38.71%), 23,370 (57.85%), and 15,986 (39.57%) returned at least one hit after the blastp analysis with nr, Araport11, RefSeq, SwissProt, and TrEMBL databases as subjects, respectively (Supplementary Table 8). Of the 40,397 predicted transcripts, 29,146 (72.2%) returned a functional annotation. Functional annotation analyses assigned InterPro domain, GO, KEGG pathway, and KEGG ortholog terms to 29,073 (72.0%), 20,580 (50.9%), 8,999 (22.3%), and 7,142 (17.7%) of the predicted transcripts, respectively (Supplementary Table 9).

### Potential candidate genes for primocane-fruiting in blackberry

In blackberry, the primocane-fruiting trait is caused by a single recessive locus that has been mapped between markers FF683693.1 RH_MEa0007aG06 and FF683518.1 RH_MEa0006aC04 in an SSR-based linkage map of the tetraploid population “Prime-Jim” × “Arapaho” (Castro *et al.* 2013). While these markers were originally placed on linkage group 7 of blackberry, it was later shown that the flanking markers and most others from linkage group 7 of the “Prime-Jim” × “Arapaho” aligned to chromosome 2 of *R. occidentalis* (VanBuren *et al.* 2016). Based on our genomic data, these markers are located on *R. argutus* chromosome Ra02 at 25,901,374–25,901,083 bp (FF683518.1 RH_MEa0006aC04) and 37,085,586–37,085,204 bp (FF683693.1 RH_MEa0007aG06). Interestingly, different loci have been found to control primocane-fruiting in raspberry (Jibran *et al.* 2019) and everbearing flowering in diploid and octoploid strawberries (Koskela *et al.* 2012; Gaston *et al.* 2013), suggesting that flowering in first-year shoots has evolved multiple times in the Rosaceae.

To explore candidate genes for the primocane-fruiting trait in blackberry, blackberry homologs of the *Arabidopsis* flowering time genes listed in FLOR-ID database were mined from the “Hillquist” genome sequence (Supplementary Table 10; Bouché *et al.* 2016). Based on blackberry gene annotations and BLAST analyses, 18 flowering gene homologs were identified within the ∼11.2 Mb primocane-fruiting locus on “Hillquist” chromosome Ra02 (Table 3). Almost half of the genes were involved in epigenetic processes that control gene expression through histone methylation, histone ubiquitinylation, small RNA processing, or as a component of nucleosome assembly. Moreover, 6 putative transcription factors and 3 photoperiodic flowering pathway genes (*LATE*, *PRR7*, *CIB4*) were identified in the primocane-fruiting locus.

**Table 3.** *Rubus argutus* flowering gene homologs identified in the primocane-fruiting locus from 25.9 to 37.1 Mb on chromosome Ra02.

*Rubus argutus* gene	Arabidopsis *thaliana* gene locus	*Arabidopsis thaliana* gene name	Effect on flowering in *A. thaliana*	Function
Ra_g7484.t1	AT3G43920.2	*DCL3*	Activator	Small RNA processing^a^
Ra_g7549.t1	AT5G48890.2	*LATE*	Repressor	C2H2-like zinc finger transcription factor^b^
Ra_g7855.t1	AT3G54560.2	*HTA11*	Repressor	Histone variant H2A.Z^c^
Ra_g8160.t1	AT4G02560.2	*LD*	Activator	Prion domain protein^d^
Ra_g8161.t1	AT4G02560.2	*LD*	Activator	Prion domain protein^d^
Ra_g8165.t1	AT5G44160.1	*IDD8*	Activator	Indeterminate domain transcription factor^e^
Ra_g8239.t1	AT4G02560.2	*LD*	Activator	Prion domain protein^d^
Ra_g8394.t1	AT5G02810.2	*PRR7*	Activator	CCT transcription factor^f^
Ra_g8513.t1	AT1G77300.1	*SDG8/ASHH2/EFS*	Repressor	Histone lysine N-methyltransferase^g^
Ra_g8554.t1	AT1G05830.4	*ATX2*	Repressor	Histone lysine N-methyltransferase^h^
Ra_g8752.t1	AT4G31120.1	*SKB1*	Activator	Histone arginine methyltransferase^i^
Ra_g8759.t1	AT3G49600.2	*UBP26*	Repressor	Ubiquitin-specific protease^j^
Ra_g8765.t1	AT3G49600.2	*UBP26*	Repressor	Ubiquitin-specific protease^j^
Ra_g8779.t1	AT5G24860.1	*FPF1*	Activator	Unknown^k^
Ra_g8780.t1	AT3G43920.2	*DCL3*	Activator	Small RNA processing^a^
Ra_g8885.t1	AT4G32980.2	*ATH1*	Repressor	Homeobox transcription factor^l^
Ra_g8936.t1	AT1G10120.2	*CIB4/BHLH74*	Activator	bHLH transcription factor^m^
Ra_g9251.t1	AT5G11530.3	*EMF1*	Repressor	PcG protein^n^

a Schmitz *et al.* (2007).

b Weingartner *et al.* (2011).

c Choi *et al.* (2007).

d Chakrabortee *et al.* (2016).

e Seo *et al.* (2011).

f Nakamichi *et al.* (2007).

g Zhao *et al.* (2005).

h Saleh *et al.* (2008).

i Wang *et al.* (2007).

j Schmitz *et al.* (2009).

k Kania *et al.* (1997).

l Proveniers *et al.* (2007).

m Liu *et al.* (2013).

n Kim *et al.* (2012).

Ten and 8 of the 18 flowering genes in the locus encoded activators and repressors of flowering in *Arabidopsis*, respectively. Floral repressors are primary candidates for primocane-fruiting because a loss-of-function mutation in a repressor could cause this trait to be recessively inherited. Among transcription factors in the locus that repress flowering, LATE is a C2H2 zinc‐finger protein that represses the expression of photoperiodic pathway genes *CO* and *FT* (Weingartner *et al.* 2011) and ATH1 is involved in the activation of *FLC* in *Arabidopsis* (Proveniers *et al.* 2007). Furthermore, many of the identified epigenetic regulators, including STG8, ATX2, UBP26, and EMF1, functioned as floral repressors in *Arabidopsis* by activating the expression of *FLC* (Zhao *et al.* 2005; Saleh *et al.* 2008; Schmitz *et al.* 2009; Kim *et al.* 2012). No clear *FLC* ortholog was found in the “Hillquist” genome assembly, but these epigenetic regulators likely regulate other targets in blackberry as observed in *Arabidopsis* (Saleh *et al.* 2008; Kim *et al.* 2012).

Other promising candidate genes identified were *PRR7* and *FD*. *PRR7* encodes a floral activator in *Arabidopsis* (Nakamichi *et al.* 2007), and a homologous gene called *BTC1* is involved in the annual to biennial transition in sugar beet (Pin *et al.* 2012). However, if PRR7 controls primocane-fruiting in blackberry, the mechanism is different from beet. In beet, recessive *btc1* alleles confer an obligatory vernalization response and postpone floral initiation into the spring of the second year (Pin *et al.* 2012), while recessive alleles of the primocane-fruiting locus cause flowering during the first year in blackberry (Lopez-Medina *et al.* 2000).

Previous studies have shown that *TFL1* encodes a strong repressor of flowering in several Rosaceous species. For example, in diploid woodland strawberry, nonfunctional *TFL1* alleles cause rapid and perpetual flowering in long day conditions (Koskela *et al.* 2012). Similarly, RNA-silencing of *TFL1* orthologs in cultivated strawberry, apple, and pear caused comparative phenotypes in these species (Flachowsky *et al.* 2012; Freiman *et al.* 2012; Koskela *et al.* 2016). Therefore, *TFL1* is also expected to play an important role in the control of flowering in blackberry, and it is a potential target of identified candidate genes. A gene encoding the bZIP transcription factor FD was identified just outside the primocane-fruiting locus in the “Hillquist” genome. Recent results show that TFL1 competes for binding to FD with floral activator FT to control common target genes in *Arabidopsis* (Zhu *et al.* 2020). Therefore, a mutation in *FD* could potentially prevent *TFL1* from repressing floral activators that are needed for floral initiation during the first season in primocane-fruiting genotypes, leading to the observed phenotype.

## Conclusions

The first high-quality chromosome-length genome assembly and annotation of the diploid blackberry *R. argutus* “Hillquist” is reported in this manuscript. Comparisons of the “Hillquist” genome with the related species *R. idaeus* (Davik *et al.* 2022) and *R. chingii* (Wang *et al.* 2021) demonstrated that the Hi-C assembly represented the majority of the genome and was of high quality. BUSCO analysis and comparisons of predicted genes with other *Rubus* genomes showed that the structural and functional annotations of the assembly were also comprehensive. Analysis of repeat content revealed that approximately 52.8% of the genome was composed of repetitive elements and that the *Gypsy* superfamily of LTR-REs accounted for the largest fractions of the genome. Developing new GBS-based maternal haplotype map of the tetraploid blackberry breeding selection A-2551TN that was highly collinear with the physical sequence of “Hillquist” demonstrated the utility of this new genome for molecular breeding applications in tetraploid fresh-market blackberries. The new “Hillquist” genome assembly and its annotation were also used to identify potential candidate genes for the economically important trait of primocane-fruiting. The “Hillquist” genome sequence and annotation presented here will assist blackberry breeders and scientists in marker development and genomic-assisted breeding and facilitate future studies of *Rubus* biology, genetics, and genomics.

## Data Availability

Raw PacBio and IsoSeq sequencing data and the genome assembly of R. argutus presented here are available at the NCBI under Bioproject ID PRJNA830911. Hi-C data are available on Bioproject PRJNA512907 (Biosample SAMN15804004; SRA SRX8934844). Illumina transcriptome data are available on Bioproject PRJEB36280 (BioSamples SAMEA6502409, SAMEA6502410, SAMEA6502411, SAMEA6502412, and SAMEA6502413). Interactive Hi-C contact maps of the “Hillquist” genome sequence assembly are available via the www.dnazoo.org website (https://tinyurl.com/2eldso37). The “Hillquist” genome assembly and annotation can also be accessed at the Genome Database for Rosaceae (https://www.rosaceae.org/Analysis/13328362; Jung *et al*. 2019) under the accession number tfGDR1056. Supplemental Material is available at figshare: https://doi.org/10.25387/g3.21375603.

## References

[jkac289-B1] Afgan E , Baker D, Batut B, van den Beek M, Bouvier D, Cech M, Chilton J, Clements D, Coraor N, Grüning BA, et al The Galaxy platform for accessible, reproducible and collaborative biomedical analyses: 2018 update. Nucleic Acids Res. 2018;46(W1):W537–W544. 29790989 10.1093/nar/gky379 PMC6030816

[jkac289-B2] Altschul S , Gish W, Miller W, Myers E, Lipman D. Basic local alignment search tool. J Mol Biol. 1990;215(3):403–410. 2231712 10.1016/S0022-2836(05)80360-2

[jkac289-B3] Andrews S. FastQC: A Quality Control Tool for High Throughput Sequence Data. 2010. http://www.bioinformatics.babraham.ac.uk/projects/fastqc.

[jkac289-B4] Bolger AM , Lohse M, Usadel B. Trimmomatic: a flexible trimmer for Illumina sequence data. Bioinformatics. 2014;30(15):2114–2120. 24695404 10.1093/bioinformatics/btu170 PMC4103590

[jkac289-B5] Bouché F , Lobet G, Tocquin P, Périlleux C. FLOR-ID: an interactive database of flowering-time gene networks in *Arabidopsis thaliana*. Nucleic Acids Res. 2016;44(D1):D1167–D1171. 26476447 10.1093/nar/gkv1054 PMC4702789

[jkac289-B6] Brůna T , Hoff KJ, Lomsadze A, Stanke M, Borodovsky M. BRAKER2: automatic eukaryotic genome annotation with GeneMark-EP+ and AUGUSTUS supported by a protein database. NAR Genomics Bioinforma. 2021;3:lqaa108. 10.1093/nargab/lqaa108 PMC7787252 33575650

[jkac289-B7] Brůna T , Lomsadze A, Borodovsky M. GeneMark-EP+: eukaryotic gene prediction with self-training in the space of genes and proteins. NAR Genomics Bioinformatics. 2020;2:lqaa026. 32440658 10.1093/nargab/lqaa026 PMC7222226

[jkac289-B8] Camacho C , Coulouris G, Avagyan V, Ma N, Papadopoulos J, Bealer K, Madden TL. BLAST+: architecture and applications. BMC Bioinformatics. 2009;10:421. 20003500 10.1186/1471-2105-10-421 PMC2803857

[jkac289-B9] Carter KA , Liston A, Bassil NV, Alice LA, Bushakra JM, Sutherland BL, Mockler TC, Bryant DW, Hummer KE. Target capture sequencing unravels *Rubus* evolution. Front Plant Sci. 2019;10:1615. 31921259 10.3389/fpls.2019.01615 PMC6933950

[jkac289-B10] Castro P , Stafne ET, Clark JR, Lewers KS. Genetic map of the primocane-fruiting and thornless traits of tetraploid blackberry. Theor Appl Genet. 2013;126(10):2521–2532. 23856741 10.1007/s00122-013-2152-3

[jkac289-B11] Chakrabortee S , Kayatekin C, Newby GA, Mendillo ML, Lancaster A, Lindquist S. Luminidependens (LD) is an *Arabidopsis* protein with prion behavior. Proc Natl Acad Sci USA. 2016;113(21):6065–6070. 27114519 10.1073/pnas.1604478113 PMC4889399

[jkac289-B12] Chin C-S , Peluso P, Sedlazeck FJ, Nattestad M, Concepcion GT, Clum A, Dunn C, O'Malley R, Figueroa-Balderas R, Morales-Cruz A, et al Phased diploid genome assembly with single-molecule real-time sequencing. Nat. Methods. 2016;13(12):1050–1054. 27749838 10.1038/nmeth.4035 PMC5503144

[jkac289-B13] Choi K , Park C, Lee J, Oh M, Noh B, Lee I. *Arabidopsis* homologs of components of the SWR1 complex regulate flowering and plant development. Development. 2007;134(10):1931–1941. 17470967 10.1242/dev.001891

[jkac289-B14] Clark JR. Primocane-fruiting blackberry breeding. HortScience. 2008;43(6):1637–1639.

[jkac289-B15] Clark JR , Moore JN, Lopez-Medina J, Finn C, Perkins-Veazie P. “Prime-Jan” ('APF-8’) and “Prime-Jim” ('APF-12’) primocane-fruiting blackberries. HortScience. 2005;40(3):852–855.

[jkac289-B16] Clark JR , Stafne ET, Hall HK, Region N, Finn CE. Blackberry breeding and genetics. Plant Breed. Rev. 2007;29:19–144.

[jkac289-B17] Daccord N , Celton J-M, Linsmith G, Becker C, Choisne N, Schijlen E, van de Geest H, Bianco L, Micheletti D, Velasco R, et al High-quality de novo assembly of the apple genome and methylome dynamics of early fruit development. Nat Genet. 2017;49(7):1099–1106. 28581499 10.1038/ng.3886

[jkac289-B18] Davik J , Røen D, Lysøe E, Buti M, Rossman S, Alsheikh M, Aiden EL, Dudchenko O, Sargent DJ. A chromosome-level genome sequence assembly of the red raspberry (*Rubus idaeus* L.). PLoS One. 2022;17(3):e0265096. 35294470 10.1371/journal.pone.0265096 PMC8926247

[jkac289-B19] Dobin A , Davis CA, Schlesinger F, Drenkow J, Zaleski C, Jha S, Batut P, Chaisson M, Gingeras TR. STAR: ultrafast universal RNA-seq aligner. Bioinformatics. 2013;29(1):15–21. 23104886 10.1093/bioinformatics/bts635 PMC3530905

[jkac289-B20] Dudchenko O , Batra SS, Omer AD, Nyquist SK, Hoeger M, Durand NC, Shamim MS, Machol I, Lander ES, Aiden AP, et al De novo assembly of the *Aedes aegypti* genome using Hi-C yields chromosome-length scaffolds. Science. 2017;356(6333):92–95. 28336562 10.1126/science.aal3327 PMC5635820

[jkac289-B22] Durand NC , Shamim MS, Machol I, Rao SSP, Huntley MH, Lander ES, Aiden EL. Juicer provides a one-click system for analyzing loop-resolution Hi-C experiments. Cell Syst. 2016;3(1):95–98. 27467249 10.1016/j.cels.2016.07.002 PMC5846465

[jkac289-B23] Edger PP , Poorten TJ, VanBuren R, Hardigan MA, Colle M, McKain MR, Smith RD, Teresi SJ, Nelson ADL, Wai CM, et al Origin and evolution of the octoploid strawberry genome. Nat Genet. 2019;51(3):541–547. 30804557 10.1038/s41588-019-0356-4 PMC6882729

[jkac289-B24] Edger PP , VanBuren R, Colle M, Poorten TJ, Wai CM, Niederhuth CE, Alger EI, Ou S, Acharya CB, Wang J, et al Single-molecule sequencing and optical mapping yields an improved genome of woodland strawberry (*Fragaria vesca*) with chromosome-scale contiguity. Gigascience. 2018;7(2):1–7. 10.1093/gigascience/gix124 PMC5801600 29253147

[jkac289-B25] Ellinghaus D , Kurtz S, Willhoeft U. LTRharvest, an efficient and flexible software for de novo detection of LTR retrotransposons. BMC Bioinformatics. 2008;9:18. 18194517 10.1186/1471-2105-9-18 PMC2253517

[jkac289-B26] Finn CE , Clark JR. Blackberry. In: Badenes ML, Byrne DH, editors. Fruit Breeding. New York (NY): Springer Science + Business Media; 2012. p. 151–190.

[jkac289-B27] Flachowsky H , Szankowski I, Waidmann S, Peil A, Tränkner C, Hanke M-V. The *MdTFL1* gene of apple (*Malus* × *domestica* Borkh.) reduces vegetative growth and generation time. Tree Physiol. 2012;32(10):1288–1301. 23022687 10.1093/treephys/tps080

[jkac289-B28] Flynn JM , Hubley R, Goubert C, Rosen J, Clark AG, Feschotte C, Smit AF. RepeatModeler2 for automated genomic discovery of transposable element families. Proc Natl Acad Sci USA. 2020;117(17):9451–9457. 32300014 10.1073/pnas.1921046117 PMC7196820

[jkac289-B29] Focke WO. Species ruborum. Monographiae Generis Rubi Prodromus. Stuttgart: Schweizerbart; 1910.

[jkac289-B30] Freiman A , Shlizerman L, Golobovitch S, Yablovitz Z, Korchinsky R, Cohen Y, Samach A, Chevreau E, Le Roux P-M, Patocchi A, et al Development of a transgenic early flowering pear (*Pyrus communis* L.) genotype by RNAi silencing of PcTFL1-1 and PcTFL1-2. Planta. 2012;235(6):1239–1251. 22203321 10.1007/s00425-011-1571-0

[jkac289-B31] Gao Y , Yang Q, Yan X, Wu X, Yang F, Li J, Wei J, Ni J, Ahmad M, Bai S, et al High-quality genome assembly of “Cuiguan” pear (*Pyrus pyrifolia*) as a reference genome for identifying regulatory genes and epigenetic modifications responsible for bud dormancy. Hortic Res. 2021;8(1):197. 34465760 10.1038/s41438-021-00632-w PMC8408243

[jkac289-B32] Gaston A , Perrotte J, Lerceteau-Köhler E, Rousseau-Gueutin M, Petit A, Hernould M, Rothan C, Denoyes B. *PFRU*, a single dominant locus regulates the balance between sexual and asexual plant reproduction in cultivated strawberry. J Exp Bot. 2013;64(7):1837–1848. 23554259 10.1093/jxb/ert047

[jkac289-B33] Germplasm Resources Information Network (GRIN) [online database] . USDA, ARS, Natl Genet Resour Progr. Beltsville (MD): National Germplasm Resources Laboratory; 2022. [accessed 2022 November 20].

[jkac289-B34] Huerta-Cepas J , Forslund K, Coelho LP, Szklarczyk D, Jensen LJ, von Mering C, Bork P. Fast genome-wide functional annotation through orthology assignment by eggNOG-mapper. Mol Biol Evol. 2017;34(8):2115–2122. 28460117 10.1093/molbev/msx148 PMC5850834

[jkac289-B35] Jibran R , Dzierzon H, Bassil N, Bushakra JM, Edger PP, Sullivan S, Finn CE, Dossett M, Vining KJ, VanBuren R, et al Chromosome-scale scaffolding of the black raspberry (*Rubus occidentalis* L.) genome based on chromatin interaction data. Hortic Res. 2018;5:8. 29423238 10.1038/s41438-017-0013-y PMC5802725

[jkac289-B36] Jibran R , Spencer J, Fernandez G, Monfort A, Mnejja M, Dzierzon H, Tahir J, Davies K, Chagné D, Foster TM, et al Two loci, *RiAF3* and *RiAF4*, contribute to the annual-fruiting trait in *Rubus*. Front Plant Sci. 2019;10:1341. 31708950 10.3389/fpls.2019.01341 PMC6824294

[jkac289-B37] Jung S , Lee T, Cheng C-H, Buble K, Zheng P, Yu J, Humann J, Ficklin SP, Gasic K, Scott K, et al 15 years of GDR: new data and functionality in the Genome Database for Rosaceae. Nucleic Acids Res. 2019;47(D1):D1137–D1145. 30357347 10.1093/nar/gky1000 PMC6324069

[jkac289-B38] Kanehisa M , Sato Y, Morishima K. BlastKOALA and GhostKOALA: KEGG Tools for functional characterization of genome and metagenome sequences. J Mol Biol. 2016;428(4):726–731. 26585406 10.1016/j.jmb.2015.11.006

[jkac289-B39] Kania T , Russenberger D, Peng S, Apel K, Melzer S. *FPF1* promotes flowering in *Arabidopsis*. Plant Cell. 1997;9(8):1327–1338. 9286110 10.1105/tpc.9.8.1327 PMC157001

[jkac289-B40] Keep E. Primocane (autumn)-fruiting raspberries: a review with particular reference to progress in breeding. J Hortic Sci. 1988;63(1):1–18.

[jkac289-B41] Kim SY , Lee J, Eshed-Williams L, Zilberman D, Sung ZR. EMF1 and PRC2 cooperate to repress key regulators of *Arabidopsis* development. PLoS Genet. 2012;8(3):e1002512. 22457632 10.1371/journal.pgen.1002512 PMC3310727

[jkac289-B42] Koskela EA , Mouhu K, Albani MC, Kurokura T, Rantanen M, Sargent DJ, Battey NH, Coupland G, Elomaa P, Hytönen T, et al Mutation in *TERMINAL FLOWER1* reverses the photoperiodic requirement for flowering in the wild strawberry *Fragaria vesca*. Plant Physiol. 2012;159(3):1043–1054. 22566495 10.1104/pp.112.196659 PMC3387692

[jkac289-B43] Koskela EA , Sønsteby A, Flachowsky H, Heide OM, Hanke M-V, Elomaa P, Hytönen T. *TERMINAL FLOWER1* is a breeding target for a novel everbearing trait and tailored flowering responses in cultivated strawberry (*Fragaria* × *ananassa* Duch). Plant Biotechnol J. 2016;14(9):1852–1861. 26940366 10.1111/pbi.12545 PMC5069601

[jkac289-B44] Kriventseva EV , Kuznetsov D, Tegenfeldt F, Manni M, Dias R, Simão FA, Zdobnov EM. OrthoDB v10: sampling the diversity of animal, plant, fungal, protist, bacterial and viral genomes for evolutionary and functional annotations of orthologs. Nucleic Acids Res. 2019;47(D1):D807–D811. 30395283 10.1093/nar/gky1053 PMC6323947

[jkac289-B45] Kuster RD , Yencho GC, Olukolu BA. NgsComposer: an automated pipeline for empirically based NGS data quality filtering. Brief Bioinform. 2021;221:1–10. 10.1093/bib/bbab092 PMC8425578 33822850

[jkac289-B47] Li Y , Wei W, Feng J, Luo H, Pi M, Liu Z, Kang C. Genome re-annotation of the wild strawberry *Fragaria vesca* using extensive Illumina-and SMRT-based RNA-seq datasets. DNA Res. 2018;25(1):61–70. 29036429 10.1093/dnares/dsx038 PMC5824900

[jkac289-B48] Liu Y , Li X, Li K, Liu H, Lin C. Multiple bHLH proteins form heterodimers to mediate CRY2-dependent regulation of flowering-time in *Arabidopsis*. PLoS Genet. 2013;9(10):e1003861. 24130508 10.1371/journal.pgen.1003861 PMC3794922

[jkac289-B49] Lomsadze A , Burns PD, Borodovsky M. Integration of mapped RNA-Seq reads into automatic training of eukaryotic gene finding algorithm. Nucleic Acids Res. 2014;42(15):e119. 24990371 10.1093/nar/gku557 PMC4150757

[jkac289-B50] Longhi S , Giongo L, Buti M, Surbanovski N, Viola R, Velasco R, Ward JA, Sargent DJ. Molecular genetics and genomics of the Rosoideae: state of the art and future perspectives. Hortic Res. 2014;1:1. 26504527 10.1038/hortres.2014.1 PMC4591673

[jkac289-B51] Lopez-Medina J , Moore JN, McNew RW. A proposed model for inheritance of primocane fruiting in tetraploid erect blackberry. J Am Soc Hortic Sci. 2000;125(2):217–221.

[jkac289-B52] Marçais G , Delcher AL, Phillippy AM, Coston R, Salzberg SL, Zimin A. MUMmer4: a fast and versatile genome alignment system. PLoS Comput Biol. 2018;14(1):e1005944. 29373581 10.1371/journal.pcbi.1005944 PMC5802927

[jkac289-B53] Meng R , Finn C. Determining ploidy level and nuclear DNA content in *Rubus* by flow cytometry. J Am Soc Hortic Sci. 2002;127(5):767–775.

[jkac289-B54] Mollinari M , Olukolu BA, Pereira GdS, Khan A, Gemenet D, Yencho GC, Zeng Z-B. Unraveling the hexaploid sweetpotato inheritance using ultra-dense multilocus mapping. G3 (Bethesda). 2020;10(1):281–292. 31732504 10.1534/g3.119.400620 PMC6945028

[jkac289-B55] Nakamichi N , Kita M, Niinuma K, Ito S, Yamashino T, Mizoguchi T, Mizuno T. *Arabidopsis* clock-associated pseudo-response regulators PRR9, PRR7 and PRR5 coordinately and positively regulate flowering time through the canonical CONSTANS-dependent photoperiodic pathway. Plant Cell Physiol. 2007;48(6):822–832. 17504813 10.1093/pcp/pcm056

[jkac289-B56] Neumann P , Novák P, Hoštáková N, MacAs J. Systematic survey of plant LTR-retrotransposons elucidates phylogenetic relationships of their polyprotein domains and provides a reference for element classification. Mob DNA. 2019;10:1. 30622655 10.1186/s13100-018-0144-1 PMC6317226

[jkac289-B57] Novák P , Neumann P, Macas J. Global analysis of repetitive DNA from unassembled sequence reads using RepeatExplorer2. Nat Protoc. 2020;15(11):3745–3776. 33097925 10.1038/s41596-020-0400-y

[jkac289-B58] Van Ooijen JW. JoinMap 4, Software for the Calculation of Genetic Linkage Maps in Experimental Populations. Wageningen (The Netherlands): Kyazma BV, 2006.

[jkac289-B59] Pin PA , Zhang W, Vogt SH, Dally N, Büttner B, Schulze-Buxloh G, Jelly NS, Chia TYP, Mutasa-Göttgens ES, Dohm JC, et al The role of a pseudo-response regulator gene in life cycle adaptation and domestication of beet. Curr Biol. 2012;22(12):1095–1101. 22608508 10.1016/j.cub.2012.04.007

[jkac289-B60] Porebski S , Bailey LG, Baum BR. Modification of a CTAB DNA extraction protocol for plants containing high polysaccharide and polyphenol components. Plant Mol Biol Rep. 1997;15(1):8–15.

[jkac289-B61] Produce Market Guide . Commodity: Blackberries; 2022. [accessed 2022 Apr 27]. https://www.producemarketguide.com/produce/blackberries.

[jkac289-B62] Proveniers M , Rutjens B, Brand M, Smeekens S. The *Arabidopsis* TALE homeobox gene *ATH1* controls floral competency through positive regulation of FLC. Plant J. 2007;52(5):899–913. 17908157 10.1111/j.1365-313X.2007.03285.x

[jkac289-B63] Rao SSP , Huntley MH, Durand NC, Stamenova EK, Bochkov ID, Robinson JT, Sanborn AL, Machol I, Omer AD, Lander ES, et al A 3D map of the human genome at kilobase resolution reveals principles of chromatin looping. Cell. 2014;159(7):1665–1680. 25497547 10.1016/j.cell.2014.11.021 PMC5635824

[jkac289-B64] Raymond O , Gouzy J, Just J, Badouin H, Verdenaud M, Lemainque A, Vergne P, Moja S, Choisne N, Pont C, et al The *Rosa* genome provides new insights into the domestication of modern roses. Nat Genet. 2018;50(6):772–777. 29713014 10.1038/s41588-018-0110-3 PMC5984618

[jkac289-B65] Roach MJ , Schmidt SA, Borneman AR. Purge Haplotigs: allelic contig reassignment for third-gen diploid genome assemblies. BMC Bioinformatics. 2018;19(1):460. 30497373 10.1186/s12859-018-2485-7 PMC6267036

[jkac289-B66] Saleh A , Alvarez-Venegas R, Yilmaz M, Le O, Hou G, Sadder M, Al-Abdallat A, Xia Y, Lu G, Ladunga I, et al The highly similar *Arabidopsis* homologs of trithorax *ATX1* and *ATX2* encode proteins with divergent biochemical functions. Plant Cell. 2008;20(3):568–579. 18375658 10.1105/tpc.107.056614 PMC2329920

[jkac289-B67] Schmieder R , Edwards R. Quality control and preprocessing of metagenomic datasets. Bioinformatics. 2011;27(6):863–864. 21278185 10.1093/bioinformatics/btr026 PMC3051327

[jkac289-B68] Schmitz RJ , Hong L, Fitzpatrick KE, Amasino RM. *DICER-LIKE 1* and *DICER-LIKE 3* redundantly act to promote flowering via repression of *FLOWERING LOCUS C* in *Arabidopsis thaliana*. Genetics. 2007;176(2):1359–1362. 17579240 10.1534/genetics.107.070649 PMC1894598

[jkac289-B69] Schmitz RJ , Tamada Y, Doyle MR, Zhang X, Amasino RM. Histone H2B deubiquitination is required for transcriptional activation of *FLOWERING LOCUS C* and for proper control of flowering in *Arabidopsis*. Plant Physiol. 2009;149(2):1196–1204. 19091875 10.1104/pp.108.131508 PMC2633843

[jkac289-B70] Seo PJ , Ryu J, Kang SK, Park CM. Modulation of sugar metabolism by an *INDETERMINATE DOMAIN* transcription factor contributes to photoperiodic flowering in *Arabidopsis*. Plant J. 2011;65(3):418–429. 21265895 10.1111/j.1365-313X.2010.04432.x

[jkac289-B71] Seppey M , Manni M, Zdobnov EM. BUSCO: assessing genome assembly and annotation completeness. Methods Mol Biol. 2019;1962:227–245. 31020564 10.1007/978-1-4939-9173-0_14

[jkac289-B72] Shulaev V , Sargent DJ, Crowhurst RN, Mockler TC, Folkerts O, Delcher AL, Jaiswal P, Mockaitis K, Liston A, Mane SP, et al The genome of woodland strawberry (*Fragaria vesca*). Nat Genet. 2011;43(2):109–116. 21186353 10.1038/ng.740 PMC3326587

[jkac289-B73] Shumate A , Salzberg SL. Liftoff: accurate mapping of gene annotations. Bioinformatics. 2021;37(12):1639–1643. 33320174 10.1093/bioinformatics/btaa1016 PMC8289374

[jkac289-B74] Smit A , Hubley R, Green P. RepeatMasker Open-4.0; 2013. http://www.repeatmasker.org.

[jkac289-B75] Sønsteby A , Heide OM. Environmental control of growth and flowering of *Rubus idaeus* L. cv. Glen Ample. Sci Hortic. 2008;117(3):249–256.

[jkac289-B76] Stanke M , Schöffmann O, Morgenstern B, Waack S. Gene prediction in eukaryotes with a generalized hidden Markov model that uses hints from external sources. BMC Bioinformatics. 2006;7:62. 16469098 10.1186/1471-2105-7-62 PMC1409804

[jkac289-B77] Strik BC , Clark JR, Finn CE, Bañados MP. Worldwide blackberry production. HortTechnology. 2007;17(2):205–213.

[jkac289-B78] Takeda F , Strik BC, Peacock D, Clark JR. Patterns of floral bud development in canes of erect and trailing blackberries. J Am Soc Hortic Sci. 2003;128(1):3–7.

[jkac289-B79] Thompson MM. Chromosome numbers of *Rubus* cultivars at the national clonal germplasm repository. HortScience. 1995;30(7):1453–1456.

[jkac289-B80] VanBuren R , Bryant D, Bushakra JM, Vining KJ, Edger PP, Rowley ER, Priest HD, Michael TP, Lyons E, Filichkin SA, et al The genome of black raspberry (*Rubus occidentalis*). Plant J. 2016;87(6):535–547. 27228578 10.1111/tpj.13215

[jkac289-B81] VanBuren R , Wai CM, Colle M, Wang J, Sullivan S, Bushakra JM, Liachko I, Vining KJ, Dossett M, Finn CE, et al A near complete, chromosome-scale assembly of the black raspberry (*Rubus occidentalis*) genome. Gigascience. 2018;7(8):1–9. 10.1093/gigascience/giy094 PMC6131213 30107523

[jkac289-B82] Velasco R , Zharkikh A, Affourtit J, Dhingra A, Cestaro A, Kalyanaraman A, Fontana P, Bhatnagar SK, Troggio M, Pruss D, et al The genome of the domesticated apple (*Malus* × *domestica* Borkh). Nat Genet. 2010;42(10):833–839. 20802477 10.1038/ng.654

[jkac289-B83] Verde I , Abbott AG, Scalabrin S, Jung S, Shu S, Marroni F, Zhebentyayeva T, Dettori MT, Grimwood J, Cattonaro F, et al; International Peach Genome Initiative. The high-quality draft genome of peach (*Prunus persica*) identifies unique patterns of genetic diversity, domestication and genome evolution. Nat Genet. 2013;45(5):487–494. 23525075 10.1038/ng.2586

[jkac289-B84] Vurture GW , Sedlazeck FJ, Nattestad M, Underwood CJ, Fang H, Gurtowski J, Schatz MC. GenomeScope: fast reference-free genome profiling from short reads. Bioinformatics. 2017;33(14):2202–2204. 28369201 10.1093/bioinformatics/btx153 PMC5870704

[jkac289-B85] Wadl PA , Olukolu BA, Branham SE, Jarret RL, Yencho GC, Jackson DM. Genetic diversity and population structure of the USDA sweetpotato (*Ipomoea batatas*) germplasm collections using GBSpoly. Front Plant Sci. 2018;9:1–13. 30186293 10.3389/fpls.2018.01166 PMC6111789

[jkac289-B86] Wang L , Lei T, Han G, Yue J, Zhang X, Yang Q, Ruan H, Gu C, Zhang Q, Qian T, et al The chromosome-scale reference genome of *Rubus chingii* Hu provides insight into the biosynthetic pathway of hydrolyzable tannins. Plant J. 2021;107(5):1466–1477. 34174125 10.1111/tpj.15394

[jkac289-B87] Wang X , Zhang Y, Ma Q, Zhang Z, Xue Y, Bao S, Chong K. SKB1-mediated symmetric dimethylation of histone H4R3 controls flowering time in *Arabidopsis*. Embo J. 2007;26(7):1934–1941. 17363895 10.1038/sj.emboj.7601647 PMC1847673

[jkac289-B88] Weingartner M , Subert C, Sauer N. LATE, a C2H2 zinc-finger protein that acts as floral repressor. Plant J. 2011;68(4):681–692. 21771123 10.1111/j.1365-313X.2011.04717.x

[jkac289-B90] Williams IH. Effects of environment on *Rubus idaeus* L. IV. Flower initiation and development of the inflorescence. J Hortic Sci. 1959;34(4):219–228.

[jkac289-B91] Wu TD , Watanabe CK. GMAP: a genomic mapping and alignment program for mRNA and EST sequences. Bioinformatics. 2005;21(9):1859–1875. 15728110 10.1093/bioinformatics/bti310

[jkac289-B92] Zdobnov EM , Apweiler R. InterProScan—an integration platform for the signature-recognition methods in InterPro. Bioinformatics. 2001;17(9):847–848. 11590104 10.1093/bioinformatics/17.9.847

[jkac289-B93] Zhang L , Hu J, Han X, Li J, Gao Y, Richards CM, Zhang C, Tian Y, Liu G, Gul H, et al A high-quality apple genome assembly reveals the association of a retrotransposon and red fruit colour. Nat Commun. 2019;10:1–13. 30940818 10.1038/s41467-019-09518-x PMC6445120

[jkac289-B94] Zhao Z , Yu Y, Meyer D, Wu C, Shen WH. Prevention of early flowering by expression of *FLOWERING LOCUS C* requires methylation of histone H3 K36. Nat Cell Biol. 2005;7(12):1256–1260. 16299497 10.1038/ncb1329

[jkac289-B95] Zhu Y , Klasfeld S, Jeong CW, Jin R, Goto K, Yamaguchi N, Wagner D. *TERMINAL FLOWER 1-FD* complex target genes and competition with *FLOWERING LOCUS T*. Nat Commun. 2020;11:5118. 33046692 10.1038/s41467-020-18782-1 PMC7550357

